# Inhibition of lncRNA NEAT1 sensitizes medulloblastoma cells to cisplatin through modulating the miR-23a-3p-glutaminase (GLS) axis

**DOI:** 10.1080/21655979.2021.2008695

**Published:** 2022-03-21

**Authors:** Jingjing Ge, Baohong Wang, Shuai Zhao, Jiaju Xu

**Affiliations:** Department of Pediatrics, The Affiliated Yantai Yuhuangding Hospital of Qingdao University, Yantai, China

**Keywords:** Cisplatin resistance, lncRNA NEAT1, glutaminase, glutamine metabolism, miR-23a-3p

## Abstract

Medulloblastoma (MB) is a commonly occurring brain malignancy in adolescence. Currently, the combination of chemotherapy with subsequent irradiation is a regular therapeutic strategy. However, high dosage of chemotherapy is associated with drug resistance and side effects. The long non-coding RNA nuclear paraspeckle assembly transcript 1 (NEAT1), which is frequently overexpressed in diverse human tumors, is correlated with worse survival rate in cancer patients. Currently, the precise roles of NEAT1 in MB and chemoresistance remain unclear. Our study aimed to investigate the biological functions of NEAT1 in cisplatin-resistant medulloblastoma. We report that NEAT1 was significantly upregulated in medulloblastoma patient specimens. Silencing NEAT1 significantly suppressed MB cell proliferation and sensitized MB cells to cisplatin. In cisplatin-resistant MB cell line, DAOY Cis R, NEAT1 expression, and glutamine metabolism were remarkably upregulated in cisplatin-resistant cells. Under low glutamine supply, cisplatin-resistant cells displayed increased cisplatin sensitivity. Bioinformatical analysis and luciferase assay uncovered that NEAT1 functions as a ceRNA of miR-23a-3p to downregulate its expressions in MB cells. Moreover, miR-23a-3p was apparently downregulated in MB patient tissues and cisplatin resistant MB cells. We identified GLS (glutaminase), a glutamine metabolism enzyme, was directly targeted by miR-23a-3p in MB cells. Rescue experiments demonstrated restoration of miR-23a-3p in NEAT1-overexpressing DAOY cisplatin resistant cells successfully overcame the NEAT1-promoted cisplatin resistance by targeting GLS. In general, our results revealed new molecular mechanisms for the lncRNA-NEAT1-mediated cisplatin sensitivity of MB.

## Introduction

Medulloblastoma (MB) is a common brain malignancy in adolescence [1]. Currently, therapeutic approaches against medulloblastoma comprise chemotherapy combined with surgery [2]. Due to the remarkable improvements over past decades, the long-term survival rate of MB patients has been increased [3]. However, the side effects and development of chemoresistance limited the clinical outcomes of medulloblastoma therapy [4]. Cisplatin (CDDP) is a platinum-based anti-cancer agent, which has been widely applied to combat diverse types of cancers [5]. Cisplatin functions to undermine the DNA repair processes by crosslinking with the purine bases of DNAs, leading to apoptosis of cancer cells [6]. Although cisplatin has achieved elevated survival rate for cancer patients, a large fragment of patients developed cisplatin resistance, which renders a major challenge for its clinical applications [7,8]. Therefore, investigating the underlying mechanisms and specifically therapeutic targets of the acquired cisplatin resistance is an urgent task.

LncRNAs (long non-coding RNAs) are classes of long (>200 nt), non-coding RNAs, which are known to be important mediators for tumor progressions by modulating their target gene expressions [9]. LncRNAs function through specific binding to DNA, RNA, or proteins [9]. Kinds of studies revealed that lncRNAs play essential roles in progressions of cancer, including tumorigenesis, tumor growth, migration, invasion, apoptosis, and chemoresistance [10], suggesting that lncRNAs are potential biomarkers and therapeutic targets. LncRNA-NEAT1 (long non-coding RNA nuclear paraspeckle assembly transcript 1), which is frequently overexpressed in diverse human tumors, is correlated with worse prognosis and survival rates of cancer patients [11]. However, the roles of NEAT1 in MB and cisplatin resistance remain unknown.

Accumulating studies uncovered that the aberrant glucose and glutamine metabolism are new cancer hallmarks [12]. Glutamine is an essential amino acid for the supplies of metabolic intermediates or building molecules for nucleic acid, fatty acid, as well as amino acid synthesis [13]. Moreover, targeting the dysregulated glutamine metabolism was known to effectively enhance chemotherapies [14]. Recent studies demonstrated that glutamine metabolism was significantly elevated in medulloblastoma, resulting in pool prognosis and survival rate [15]. However, the roles and precise mechanisms of NEAT1 in regulating glutamine metabolism in chemosensitivity of medulloblastoma remain unclear.

In this study, we hypothesized that blocking NEAT1 could reverse the cisplatin resistance of MB cells. Thus, we aimed to investigate the roles of NEAT1 in regulating glutamine metabolism and cisplatin resistance of MB. Cisplatin-resistant MB cell line was established. The downstream targets of NEAT1 will be identified and validated. Our study will provide new molecular mechanisms for the NEAT1-mediated cisplatin resistance of MB, contributing to the development of the lncRNA-based therapeutic approaches against chemoresistant medulloblastoma.

## Materials and methods

### Patient sample collection

A total of 40 cases of medulloblastoma patients who were clinically diagnosed with medulloblastoma from January 2015 to January 2019 at the Affiliated Yantai Yuhuangding Hospital of Qingdao University were included in this study. Specimen was collected by surgery and immediately placed in liquid nitrogen for further analysis. Medulloblastoma was diagnosed and classified according to the desmoplastic/nodular subtype, classic subtype, large cell/anaplastic, or extensive nodularity subtype. Among the patients, 23 of them were male and 17 patients were female. Twenty nine of them were diagnosed with classic MB, six of them were desmoplastic MB, and five of them were other types. In addition, 26 patients were in M0 metastatic stage, 7 patients were in M1, and the rest of them were in non-metastatic or M2 stage. No patient received chemo- or/and radio-therapies before enrolled in this study. Protocols were approved by the Institutional Review Board of the Affiliated Yantai Yuhuangding Hospital of Qingdao University. All participants signed their full consent to participate, and a written consent form was obtained from each patient.

### Cell culture and reagents

Human medulloblastoma cell lines DAOY, D341, and UW228 were obtained from the Cell Bank of the Chinese Academy of Sciences (Shanghai, China). Cells were cultured in DMEM (Dulbecco’s Modified Eagle Medium) (Invitrogen, Carlsbad, CA, USA) supplied with 10% FBS (fetal bovine serum) (Invitrogen, Carlsbad, CA, USA), 1x streptomycin, and 1x penicillin G (Thermofisher, USA) with 5% CO_2_ at 37°C. Cisplatin resistant medulloblastoma cell line DAOY was selected by treating cells with gradient concentrations of cisplatin for 2 months. Rabbit anti-GLS and β-actin antibodies were obtained from Cell Signaling Technology (#49,363, #4970, Danvers, MA, USA).

### Bioinformatics analysis

The noncoding RNAs and miRNA-mRNA associations were analyzed from starBase of ENCORI http://starbase.sysu.edu.cn/ [16].

### Transfections of plasmid DNA, siRNA and miRNAs

DAOY, D341, or UW228 cells were seeded onto six-well plates at 3 × 10^5^ per well for 24 hours. Transfections were conducted using kits Lipofectamine 2000 (Invitrogen, Carlsbad, CA, USA). DNA vector was transfected at 2 µg/well. siRNA or miRNAs was transfected at 25 nM for 48 hrs.

### Quantitative real-time PCR

RNAs from human MB tissues and cells were extracted by TRIzol kit (Thermo Fisher) according to the manufacturer’s instruction. The concentration and quality of extracted RNAs were examined by a Nanodrop 2000 Spectrophotometer (Thermo Fisher). cDNAs were synthesized from 1 µg RNA samples using the PrimeScript RT Master Mix Perfect Real Time (TaKaRa, Shiga, Japan). The qRT-PCR (quantitative real-time PCR) reaction of lncRNA NEAT1 was conducted by the SYBR Green qPCR Master Mix (Thermo Fisher). qRT-PCR reaction of miRNA was performed using a SYBR premix Ex Taq kit (Takara, Dalian, China). β-actin and human U6 were housekeeping controls for lncRNA NEAT1, mRNA, and miR-23a-3p. qRT-PCR reaction conditions were as follows: 94°C for 30 s, 55°C for 30 s, and 72°C for 90 s, for totally 35 cycles. The relative expressions of genes were analyzed by 2^−ΔΔCt^ method [17].

### Dual-luciferase reporter assay

Wild type (WT) and mutated (Mut) GLS 3ʹUTR or NEAT1 were amplified by PCR and inserted into the pGL3-control luciferase vectors (Promega). DAOY and D341 cells were plated onto 24-well plates for 24 h. Cells were transfected with control miRNA or miR-23a-3p and WT or Mut NEAT1 or GLS 3ʹUTR by Lipofectamine 2000 (Invitrogen). Cells were harvested, and the luciferase activities were measured by a Dual-Luciferase reporter assay system (Promega). The relative firefly luciferase activity was normalized to renilla luciferase activity.

### RNA pull-down assay

Sense NEAT1 and antisense NEAT1 probes were labeled with biotin from the RiboBio Co. Ltd (Guangzhou, China). Probes were incubated with MB cell lysates for 2 h, followed by incubation with Streptavidin-coupled agarose beads (Thermo Fisher). The miR-23a-3p in the RNA–RNA complex was washed out and collected. The amount of miR-23a-3p in the complex was examined by qRT-PCR.

### Measurement of glutamine metabolism

The glutamine metabolism was assessed by glutamine uptake and glutaminase activity assays, which were measured by Glutamine Assay kit (Colorimetric) (ab197011, Abcam) and a Glutaminase (GLS) Activity Assay Kit (# K455-100, BioVision, Milpitas, CA, USA). The relative glutamine uptake and GLS activity were calculated by the ratio of the readings from the treatment group to those from the control cells.

### Colony formation assay

Human medulloblastoma cells were treated with cisplatin or cultured with normal or low glutamine supply were seeded onto 12-well plates. Cells were then cultured at regular conditions for 10 days to form colonies that were stained with crystal violet (0.1%) for 5 mins at room temperature. The survival colonies were examined under microscopy. Cell accumulation with over 50 was counted as a colony.

### Cell viability

Cell viability of MB cells in response to cisplatin treatment and growth was assessed using MTT (3-(4,5-Dimethylthiazol-2-yl) assay (Beyotime, Nantong, China). In brief, cells (8 × 10^3^ cells/well) were seeded onto 96-well plate. Following treatment, the medium was refreshed, and 20 μL MTT reagent (5 mg/mL) was added to each well followed by incubation for 4 h. Then, 150 μL DMSO (dimethyl sulfoxide) was added into the wells to dissolve the formed formazan. Absorbance was used with a microplate reader (Bio-Rad) measured at 450 nm. Relative cell viability was calculated by the ratio of the readings from the treatment group to those from the control cells.

### Flowcytometry analysis

Cell apoptosis rates were detected by FITC Annexin V Apoptosis Detection Kit I (BD Biosciences) according to the manufacturer’s instruction. After cisplatin treatments, 5 × 10^5^ cells per experiment were collected and centrifuged at low speed (2000 rpm). Cells were then resuspended in a binding buffer (300 mL) from the kits. Annexin V FITC was added and mixed, followed by adding PI (propidium iodide) solution (5 mL) for 15 minutes incubation in the dark. The fluorescence from stained cells was analyzed using a FACS Calibur Flow Cytometer (BD Biosciences).

### Western blot analysis

MB cell lines were harvested and lysed by RIPA buffer (Beyotime, Nantong, China) supplied with protease inhibitor cocktail (1x) (Sigma-Aldrich). Protein concentrations were measured by Bradford assay. An equal amount of protein (40 μg) was loaded onto a 10% SDS-PAGE gel. Proteins in the gel were electro-transferred to PVDF (polyvinylidene difluoride) membranes, which were blocked with 5% BSA for 1 h. Membrane was incubated with diluted primary antibodies (1:1000) in PBST at 4°C for overnight. After washing with PBST for 3 times of 5 min each, membranes were incubated with secondary antibody at room temperature for 1 h. The proteins from membranes were detected by Hyperfilm-ECL kits (GE Healthcare Biosciences). β-actin was a loading control.

## Statistical analysis

Statistical significance of data from the experiment was analyzed using Prism 6.0 (GraphPad Software, La Jolla, CA, USA). Data were expressed as means ± SD (standard deviation). Comparisons between the two groups were analyzed using the unpaired Student’s t-test. Data from three groups or more were analyzed using one-way ANOVA. Differences with a two-sided *p* value < 0.05 were considered to be statistically significant.

## Results

### NEAT1 is positively associated with medulloblastoma and contributes to cisplatin resistance

**Figure 1.**
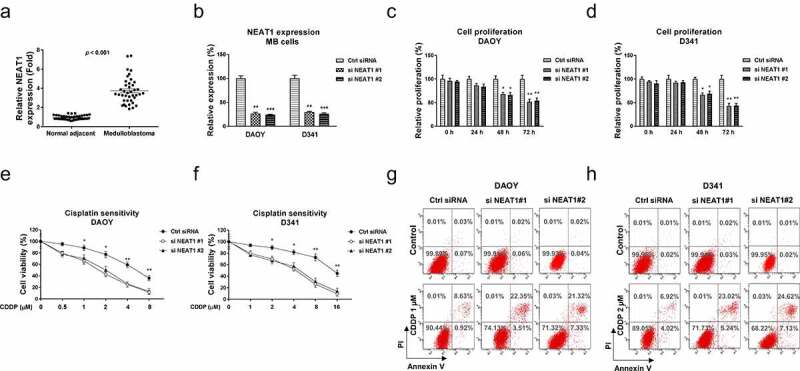
NEAT1 is elevated in medulloblastoma and leads to cisplatin resistance. (a) Expressions of NEAT1 were detected in MB tumor (n = 40) and adjacent non-tumor specimen (n = 40) by q-RT-PCR. (b) NEAT1 was silenced in DAOY and D341 cells by NEAT1 siRNA transfection. (c, d) Cell proliferation assays were performed in DAOY and D341 cells with control siRNA or NEAT1 siRNA in 0, 24, 48 and 72 hours. (e, f) The above transfected cells were exposed to cisplatin at the indicated concentrations for forty-eight hours, cells viability was examined by MTT assay and (g, h) Annexin V apoptosis assay. *, *p* < 0.05; **, *p* < 0.01; *** *p* < 0.001.

Accumulating studies revealed that NEAT1 functions as an oncogenic molecule in various cancers. Thus, we aimed to investigate the roles of NEAT1 in regulating glutamine metabolism and cisplatin resistance of MB. Cisplatin-resistant MB cell line was established. The downstream targets of NEAT1 will be identified and validated. To explore the clinical roles of lncRNA NEAT1 in medulloblastoma, we analyzed the NEAT1 expressions in 40 cases of medulloblastoma patients and adjacent tissues using qRT-PCR. The transcription level of NEAT1 was significantly increased in MB tissues compared with the paired noncancerous tissues (Figure 1a), suggesting NEAT1 plays an oncogenic role in medulloblastoma. Moreover, we analyzed NEAT1 expressions in 40 MB patients, which were classified as four groups. NEAT1 was significantly upregulated in Group 3 and Group 4 MB patients compared with those from WNT group (Fig. S1). In SHH group, NEAT1 did not significantly change (Fig. S1). To assess the functions of NEAT1 in medulloblastoma, NEAT1 was silenced in three MB cell lines, DAOY, D341 (Figure 1b) and UW228 (Fig. S2A). As we expected, silencing NEAT1 effectively inhibited cell proliferation rates of MB cells (Figure 1c, 1d, S2B). Forty-eight and seventy-two hours post transfection, DAOY, D341, and UW228 cells consistently displayed impaired cell growth. We further examined the roles of NEAT1 in cisplatin sensitivity of medulloblastoma cells. MB cells were transfected with control siRNA or NEAT1 siRNA for 48 h, followed by treatments with cisplatin at elevated concentrations. Cell viability assays demonstrated that MB cells with lower NEAT1 expression showed increased cisplatin sensitivity (Figure 1e, 1f, S2C). The cisplatin IC50s of DAOY, D341, and UW228 were 5.2 µM, 14.8 µM, 13.4 µM, respectively. Silencing NEAT1 significantly decreased the IC50s of DAOY, D341, and UW228 cells to 1.6 µM, 4.1 µM, and 3.8 µM (Figure 1e, 1f, S2C). Consistent results were obtained from Annexin V cell apoptosis assays (Figure 1g, 1h). Taken together, these results clearly reveal an oncogenic role of NEAT1, which is positively associated with cisplatin resistance in medulloblastoma.

### Cisplatin resistant medulloblastoma cells display elevated NEAT1 expression and glutamine metabolism

**Figure 2.**
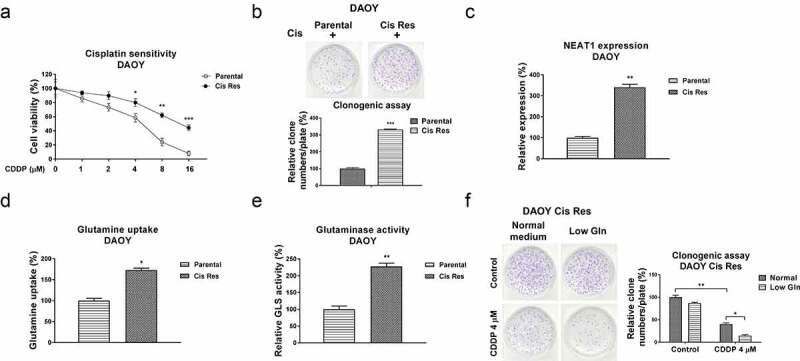
Associations of NEAT1 and glutamine metabolism with cisplatin resistance in MB cells. (a) DAOY parental and cisplatin resistant cells were treated with cisplatin at 0, 1, 2, 4, 8 or 16 µM for forty-eight hours, cell responses to cisplatin were examined by MTT assay. (b) DAOY parental and Cis Res cells were exposed to cisplatin, survival cells were detected by clonogenic assay. (c) Expressions of NEAT1 in DAOY parental and Cis Res cells were determined by qRT-PCR. (d) Glutamine uptake and (e) Glutaminase activity were determined in DAOY parental and cisplatin resistant cells. (f) DAOY Cis Res cells were growth under normal or low glutamine condition, cells were then exposed to cisplatin for 48 hours. Clonogenic assay was performed. *, *p* < 0.05; **, *p* < 0.01; ***, *p* < 0.001.

In view of the positive association between NEAT1 and cisplatin resistance in medulloblastoma, we evaluated the underlying cellular mechanisms of the NEAT1-promoted cisplatin resistance. An increasing number of studies have revealed that the increased glutamine consumption of cancer cells plays critical roles in tumor development [13–15]. Furthermore, inhibiting glutamine metabolism was approved to effectively inhibit cancer cell progressions by blocking specific regulators or catalytic enzymes in glutamine metabolism pathway [15]. We thus evaluated whether the dysregulated glutamine metabolism contributed to cisplatin resistance of medulloblastoma. Two cisplatin resistant MB cell lines were established from DAOY and D341 parental cells by exposing cells with elevated concentrations of cisplatin. As shown in Figure 2a, S3A, resistance of selected cells was characterized. DAOY and D341 cisplatin-resistant cells exhibited better survival rates under cisplatin treatments compared with those from parental cells (Figure 2a, S3A). Cell viability assay indicated the IC50s of DAOY and D341 cisplatin-resistant cells jumped to 14.8 µM and 36.2 µM, which are significantly higher than those of DAOY and D341 parental cells (4.08 µM and 13.4 µM) (Figure 2a, S3A). Consistent results were detected from clonogenic assay which showed more survival colonies in cisplatin-resistant cells than those in DAOY and D341 parental cells treated with the same concentration of cisplatin (Figure 2b, S3A). Subsequently, the expressions of lncRNA NEAT1 from parental and CDDP resistant cells were compared using qRT-PCR. Expectedly, NEAT1 was apparently overexpressed in cisplatin-resistant cells (Figure 2c, S3B). In addition, the glutamine uptake and glutaminase activity of cisplatin resistant MB cells were apparently increased (Figure 2d, 2e, S3C, S3D). Under low glutamine, CDDP resistant cells showed lower survival rate with cisplatin treatments (figure 2f), indicating cisplatin-resistant medulloblastoma cells showed a glutamine addictive phenotype. In summary, the above results revealed a positive correlation between NEAT1 and glutamine metabolism in cisplatin resistant MB cells.

### NEAT1 sponges miR-23a-3p to suppress its expression in medulloblastoma cells

**Figure 3.**
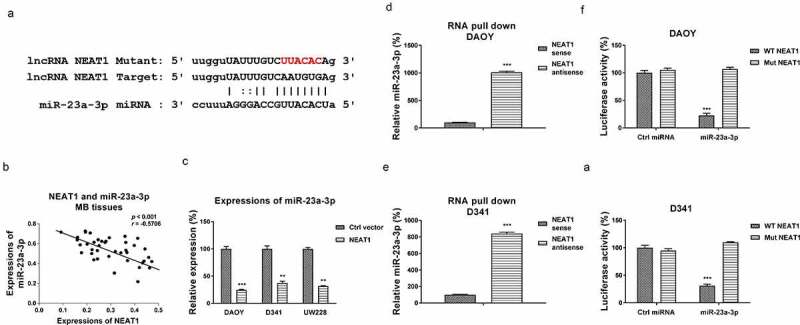
NEAT1 sponges miR-23a-3p and negatively regulates its expression. (a) The NEAT1 and miR-23a-3p interactions was predicted from starBase.com. (b) Expressions of NEAT1 and miR-23a-3p in MB tissues were analyzed by Pearson’ correlation coefficient analysis. (c) DAOY, D341 and UW228 cells were transfected with control vector or NEAT1 overexpression vector. miR-23a-3p expressions were examined by qRT-PCR. (d) DAOY and (e) D341 cells were subjected to biotin-labeled RNA pull-down assay. Amount of miR-23a-3p was determined by qRT-PCR. (f) Dual-luciferase vector with insertion of WT- or Mut- NEAT1 was co-transfected with miR-23a-3p or control miRNA into DAOY and (g) D341 cells. Luciferase activities were determined. *, *p* < 0.05; **, *p* < 0.01; ***, *p* < 0.001.

We then investigated the underlying mechanisms of the NEAT1-promoted cisplatin resistance. Accumulating evidence uncovered that lncRNAs function through suppressing their downstream target miRNAs as molecular sponges, resulting in de-repression of the miRNA-targeted mRNAs [18]. To explore the miRNA targets of NEAT1, we searched the non-coding RNA database, starBase2.0. Intriguingly, miR-23a-3p, which was reported to function as a tumor suppressive miRNA via targeting glutamine metabolism in multiple cancers, contained NEAT1 binding sites (Figure 3a). We then evaluated the relevance of miR-23a-3p and NEAT1 in medulloblastoma. Expressions of miR-23a-3p and NEAT1 were examined by Pearson’s correlation coefficient analysis. Results in Figure 3b illustrates a remarkably negative correlation between miR-23a-3p and NEAT1 in MB patient specimens, indicating that NEAT1 inhibited miR-23a-3p expression. To test this, DAOY, D341, and UW228 cells were transfected with control vector or NEAT1 overexpression plasmid. Expectedly, NEAT1 overexpression markedly downregulated miR-23a-3p expression in MB cells (Figure 3c). Consequently, the association between miR-23a-3p and NEAT1 was validated by RNA pull-down assay, which demonstrated that the amount of miR-23a-3p was enriched in the antisense NEAT1-miR-23a-3p complex (Figure 3d, 3e). To verify the binding of NEAT1 on miR-23a-3p, DAOY and D341 cells were co-transfected with luciferase plasmid, which were inserted with wild-type NEAT1 (WT-NEAT1) or mutant NEAT1 (Mut-NEAT1) plus miR-23a-3p or control miRNAs. Expectedly, cells with co-transfection of WT-NEAT1 plus miR-23a-3p precursor displayed significantly attenuated luciferase activities compared with those with Mut-NEAT1 plus control miRNAs or miRNA-23a-3p transection (figure 3f, 3g). Summarily, these results validated a NEAT1-miR-23a-3p ceRNA complex in MB cells.

### miR-23a-3p blocks glutamine metabolism and sensitizes medulloblastoma cells to cisplatin

**Figure 4.**
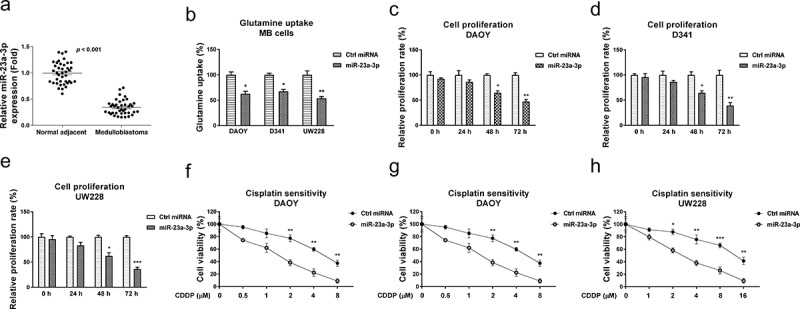
miR-23a-3p is suppressed in medulloblastoma and sensitizes MB cells to cisplatin. (a) miR-23a-3p expression levels were detected in MB tumor (n = 40) and adjacent non-tumor specimen (n = 40) by q-RT-PCR. (b) DAOY, D341 and UW228 cells were transfected with control miRNA or miR-23a-3p precursor for 48 hours, glutamine uptake was determined. (c) DAOY, (d) D341 and (e) UW228 cells were transfected with miR-23a-3p or control miRNAs, cell growth rates at 0, 24, 48 and 72 hours were determined by MTT assay. (F, G, H) The above transfected cells were exposed to cisplatin at the indicated concentrations for forty-eight hours, cells viability was examined by MTT assay. *, *p* < 0.05; **, *p* < 0.01; *** *p* < 0.001.

To investigate the biological roles of miR-23a-3p in MB cells, we compared the miR-23a-3p expressions in 40 cases of medulloblastoma patients and their matched adjacent normal tissues by qRT-PCR. Expression of miR-23a-3p was significantly downregulated in MB tissues compared with noncancerous tissues, indicating miR-23a-3p acts as a tumor suppressive miRNA in medulloblastoma (Figure 4a). Moreover, we analyzed miR-23a-3p expressions in 40 MB patients, which were classified as four groups. miR-23a-3p was significantly downregulated in Group 3 and Group 4 MB patients compared with those from WNT group (Fig. S4). In SHH group, miR-23a-3p did not significantly change (Fig. S4). To assess the functions of miR-23a-3p in the glutamine metabolism and chemoresistance of medulloblastoma cells, miR-23a-3p was transfected in three MB cell lines. The overexpression of miR-23a-3p effectively suppressed glutamine consumption (Figure 4b) and cell proliferation (Figure 4c, 4d, 4e) of MB cells. In addition, MB cells with higher miR-23a-3p expressions showed significantly increased cisplatin sensitivity (figure 4f, 4g, 4h). These results reveal miR-23a-3p acts as a tumor suppressive molecule, resulting in sensitizing medulloblastoma cells to cisplatin.

### miR-23a-3p sensitizes medulloblastoma cells to cisplatin via direct targeting glutaminase

**Figure 5.**
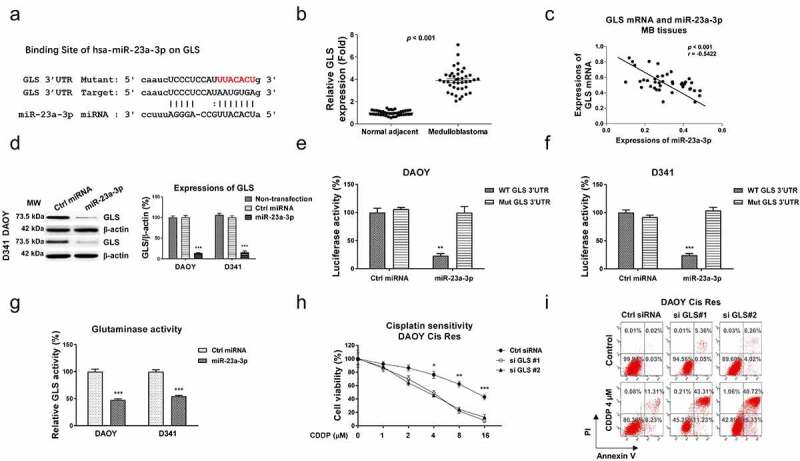
miR-23a-3p directly targets the 3ʹUTR of GLS cells. (a) Bioinformatics analysis of potential miR-23a-3p binding sites on 3ʹUTR of GLS. (b) GLS mRNA expression levels were detected in MB tumor (n = 40) and adjacent non-tumor specimen (n = 40) by q-RT-PCR. (c) Expressions of GLS and miR-23a-3p in MB tissues was analyzed by Pearson’ correlation coefficient analysis. (d) DAOY and D341 cells were transfected with control miRNA or miR-23a-3p precursor. GLS protein expressions were determined by Western blot. (e) A dual-luciferase reporter plasmid containing WT-GLS or Mut-GLS was co-transfected into DAOY and (f) D341 cells with miR-23a-3p or control miRNAs. Luciferase activities were determined. (g) Glutaminase activities were detected in MB cells without or with miR-23a-3p overexpression. (h) DAOY cisplatin resistant cells were transfected with control siRNA or two GLS siRNAs. Cells were exposed to cisplatin at the indicated concentrations. Cell viability in response to cisplatin was examined by MTT assay and (i) Annexin V apoptosis assay. *, *p* < 0.05; **, *p* < 0.01; ***, *p* < 0.001.

Since studies have uncovered that microRNAs bind to the 3ʹUTR of their target mRNAs, resulting in blocking mRNA expressions [19]. The potential targets of miR-23a-3p in MB were analyzed from the non-coding RNA database, starBase2.0. The 3ʹUTR of GLS, which catalyzes the glutamate to glutamine reaction in the glutamine metabolism pathway, contains conserved miR-23a-3p binding sites (Figure 5a). Expectedly, GLS mRNA expressions were significantly upregulated in MB patient tissues (Figure 5b). The GLS expressions in four subgroups of MB from the published database (GSE85217) were analyzed. Results in figure S5A showed that compared with WNT group, GLS expression in SHH group was slightly decreased. However, expressions of GLS in Group 3 and Group 4 were apparently upregulated. In addition, from 40 MB patients that were classified as four groups. GLS was significantly upregulated in Group 3 and Group 4 MB patients compared with those from WNT group (Fig. S5B). In SHH group, GLS expression did not significantly change (Fig. S5B). To assess the clinical correlation of the miR-23a-3p-GLS network in MB, Pearson’s correlation coefficient analysis was performed. Results in Figure 5c demonstrated that MB patients with higher GLS mRNA expressions were significantly correlated with lower miR-23a-3p levels, indicating miR-23a-3p and GLS were negatively correlated in medulloblastoma. Consequently, miR-23a-3p was overexpressed in DAOY, and D341 cells to examine whether GLS protein expression could be blocked by miR-23a-3p. Results from Figure 5d demonstrated that overexpression of miR-23a-3p significantly suppressed GLS protein expressions in MB cells. To verify whether miR-23a-3p could directly target GLS mRNA, luciferase plasmid with insertion of wild-type 3ʹUTR of GLS (WT-GLS) or miR-23a-3p binding site mutant GLS 3ʹUTR (Mut-GLS) plus miR-23a-3p or control miRNAs were co-transfected into MB cells. As expected, the luciferase activity of WT-GLS was significantly suppressed by exogenous overexpression of miR-23a-3p (Figure 5e, 5f). However, the luciferase activities were not significantly regulated in cells with control miRNAs or miR-23a-3p plus Mut-GLS vector co-transfection (Figure 5e, 5f). Given that GLS, which was positively correlated with MB progressions, was blocked by miR-23a-3p, we thus hypothesized inhibiting GLS could lead to cisplatin sensitization. *In vitro* assay demonstrated that the glutaminase activities of MB cells were effectively suppressed by miR-23a-3p overexpression (Figure 5g). Furthermore, DAOY and D341 cisplatin resistant cells with GLS knockdown by siRNA displayed increased cisplatin sensitivity compared with that from control transfected cells (Figure 5h, 5i, S6A, S6B). Consistently, DAOY and D341 cisplatin resistant cells treating with GLS inhibitor, BPTES displayed significantly increased cisplatin sensitivity (Fig. S7A, S7B). Summarily, these functional results demonstrated miR-23a-3p directly targets GLS in MB cells, leading to cisplatin sensitization.

**Figure 6.**
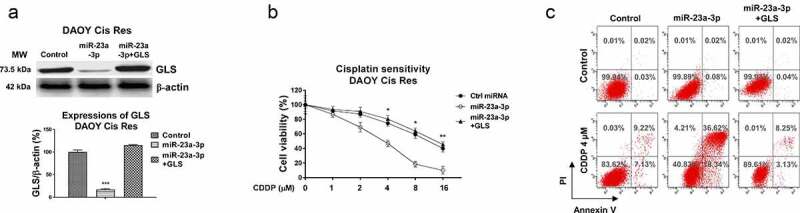
miR-23a-3p sensitizes MB cells to cisplatin via targeting GLS. (a) Control, miR-23a-3p or miR-23a-3p plus GLS overexpression vector was transfected into DAOY Cis Res cells. the GLS protein expressions were determined by Western blot. (b, c) The transfected DAOY Cis Res cells were exposed to cisplatin at variant cisplatin concentrations Cell viability in response to cisplatin was determined by MTT assay Annexin V apoptosis assay. *, *p* < 0.05; **, *p* < 0.01; ***, *p* < 0.001.

To validate whether the miR-23a-3p-modulated cisplatin sensitization was via downregulating GLS, we designed and conducted rescue experiments. DAOY Cis Res cells were transfected with control miRNAs, miR-23a-3p, or miR-23a-3p plus GLS overexpression vector. MiR-23a-3p plus GLS co-transfection effectively recovered GLS protein levels (Figure 6a). Expectedly, the rescue of GLS in miR-23a-3p-overexpressing DAOY Cis Res cells successfully recovered the cisplatin-resistant phenotypes (Figure 6b, 6c). In summary, these results validated that the miR-23a-3p-promoted cisplatin sensitization of MB cells was through targeting GLS.

### The NEAT1-promoted cisplatin resistance of medulloblastoma cells is through modifying the miR-23a-3p-GLS axis

**Figure 7.**
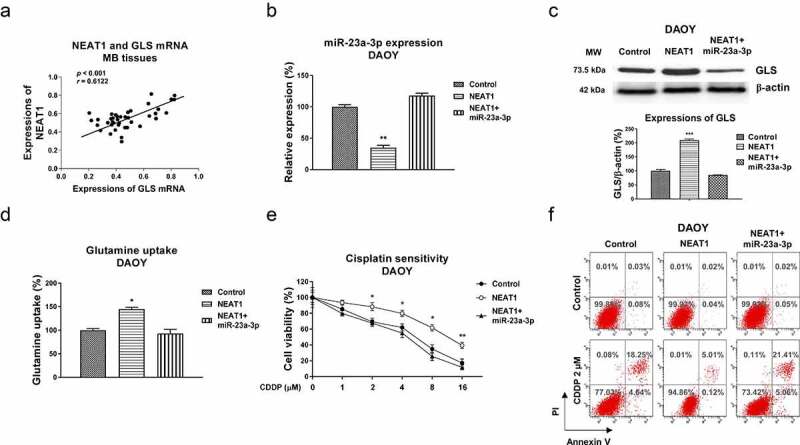
The NEAT1-miR-23a-3p-GLS axis in cisplatin resistance of MB cells. (a) Expressions of NEAT1 and GLS in MB tissues were analyzed by Pearson’ correlation coefficient analysis. (b) DAOY Cis Res cells were transfected with control, NEAT1 or NEAT1 plus miR-23a-3p, expressions of miR-23a-3p were determined by qRT-PCR and (c) Western blot. (d) Glutamine uptake was measured in the above transfected MB cells. (e) Control vector, NEAT1 or NEAT1 plus miR-23a-3p was transfected into DAOY Cis Res cells, which were treated with cisplatin at the indicated concentrations, cell viability and (f) apoptosis were examined by MTT and Annexin V assay, respectively. *, *p* < 0.05; **, *p* < 0.01; ***, *p* < 0.001.

Our above results revealed that NEAT1 promoted cisplatin resistance and sponged the miR-23a-3p-GLS pathway. Furthermore, Pearson’s correlation coefficient analysis illustrated a significantly positive correlation between NEAT1 and GLS mRNA expressions in MB patient tissues (Figure 7a). To evaluate whether the NEAT1 attenuated cisplatin sensitivity was through the miR-23a-3p-GLS axis. DAOY Cis Res cells were transfected with control vector, NEAT1, or NEAT1 with miR-23a-3p. Transfection of NEAT1 effectively blocked miR-23a-3p and de-repressed GLS protein level (Figure 7b. 7 C). Moreover, the NEAT1-regulated phenotypes were reversed by miR-23a-3p restoration (Figure 7b, 7c), indicating the NEAT1-promoted GLS expression was through miR-23a-3p inhibition. Furthermore, the NEAT1 and miR-23a-3p-co-transfected DAOY cisplatin resistant cells with showed effective re-suppression of glutamine uptake (Figure 7d) and cisplatin sensitivity compared with NEAT1 overexpression alone (Figure 7e, 7f). In summary, these data elucidated that NEAT1 modulates miR-23a-3p-GLS axis to promote the glutamine metabolism of MB cells, resulting in cisplatin resistance.

## Discussion

Medulloblastoma, a commonly malignant tumor, is one of the leading causes of nervous system dysfunction-related mortalities in children [1–3]. The high invasiveness and metastasis through cerebrospinal fluid of medulloblastoma lead to irreversible damages to the nervous system [1–3]. Although therapeutic approaches in MB patients have been improved over the past decades, side effects and chemoresistance grievously limit the therapeutic outcomes and survivals of medulloblastoma patients. Thus, investigation of the underlying molecular mechanisms and effective chemotherapeutic drugs are urgent tasks for anti-medulloblastoma treatment. This study first uncovered a lncRNA-based molecular mechanism of cisplatin resistance in MB, presenting lncRNA-NEAT1 as a potentially therapeutic target.

Studies have uncovered that lncRNAs are critical regulators in chemoresistance of the central nervous system-related diseases, such as medulloblastoma and glioma [20], suggesting lncRNAs are biomarkers and potentially therapeutic targets against medulloblastoma. NEAT1 is frequently regulated in diverse human diseases [21–23]. High NEAT1 is positively associated with a worse prognosis and low survival of cancer patients [24]. However, the biological roles of lncRNA NEAT1 in medulloblastoma have not been unveiled. Here, we demonstrated that NEAT1 was significantly overexpressed in medulloblastoma specimens compared to matched normal brain tissues, suggesting a positive association between NEAT1 and MB progression. In the established DAOY Cis Res cells, expression of NEAT1 was remarkebly upregulated compared with DAOY parental cells. Moreover, silencing NEAT1 effectively sensitized MB cells to cisplatin.

We further explored the underlying molecular targets, which contribute to the oncogenic phenotypes, of NEAT1 in MB. Since mounting studies indicated that lncRNAs function through regulating target miRNA expressions by formation of a ceRNA/miRNA network [18], we validated miRNA-23a-3p as a NEAT1-sponged miRNA in MB cells by luciferase and RNA pull-down assays. The overexpression of NEAT1 remarkebly blocked miR-23a-3p in MB cells. MiR-23a-3p was reported as a tumor suppressor, which was frequently downregulated in various cancers [25–28]. Results from qRT-PCR showed that expression of miR-23a-3p was significantly attenuated in MB patient tissues. Overexpression of miR-23a-3p inhibited cell growth and increased cisplatin sensitivity, indicating miR-23a-3p could be an anti-medulloblastoma agent. Furthermore, we searched for potential mRNA targets of miR-23a-3p from a non-coding RNA database. Consistent with previous reports [29], the luciferase assay illustrated miR-23a-3p specifically bonds on the 3ʹUTR of GLS, which is a critical enzyme catalyzing glutamine metabolism. Importantly, rescue experiment validated that the miR-23a-3p-promoted cisplatin sensitivity was through targeting GLS. Although the NEAT1-miR-23a-3p association has been reported in other cancers [30–33], our data provide a first insight into the roles of NEAT1-miR-23a-3p-GLS axis in cisplatin sensitivity of MB cells.

Cancer cells displayed dysregulated cellular metabolism for rapid proliferation, a phenomenon is called ‘Warburg effect’ [25–34]. Reprogramming of cellular metabolism including glucose and glutamine metabolism renders cancer cells advantages against chemotherapy [13,35,36]. Glutamine metabolism as well as GLS have been reported to be apparently elevated in diverse cancers and are positively associated with the tumor malignancies [13]. Our findings revealed the glutamine consumption and glutaminase activity were remarkably upregulated in cisplatin resistant MB cells. Moreover, cisplatin resistant MB cells exhibited a glutamine addictive phenotype that under low glutamine supply, DAOY Cis Res cells were more sensitive to cisplatin, suggesting blocking glutamine metabolism pathway could effectively overcome chemoresistance of MB. We demonstrated that NEAT1-mediated cisplatin resistance was via modulating the miR-23a-3p-GLS axis in MB by a mechanism rescue experiment that co-transfection of NEAT1 and miR-23a-3p recovered cisplatin sensitivity through modulating GLS. This study still has limits that these *in vitro* conclusions need *in vivo* mice experiments to validate.

## Conclusions

In summary, we demonstrate that NEAT1 functions as an oncogene in MB cell proliferation and drug resistance. Mechanically, NEAT1 sponges miR-23a-3p to form a ceRNA network to regulate expression of the miR-23a-3p target, GLS, leading to cisplatin resistance in medulloblastoma. Our study will provide novel strategies for the treatment of chemoresistant medulloblastoma.

## Supplementary Material

Supplemental Material
