# Supplementary figures and images for: Inhibition of lncRNA NEAT1 sensitizes medulloblastoma cells to cisplatin through modulating the miR-23a-3p-glutaminase (GLS) axis
Source: Bioengineered. 2022 Mar 21;13(3):7670–82. doi: 10.1080/21655979.2021.2008695 (PMC9208477; doi:10.1080/21655979.2021.2008695)

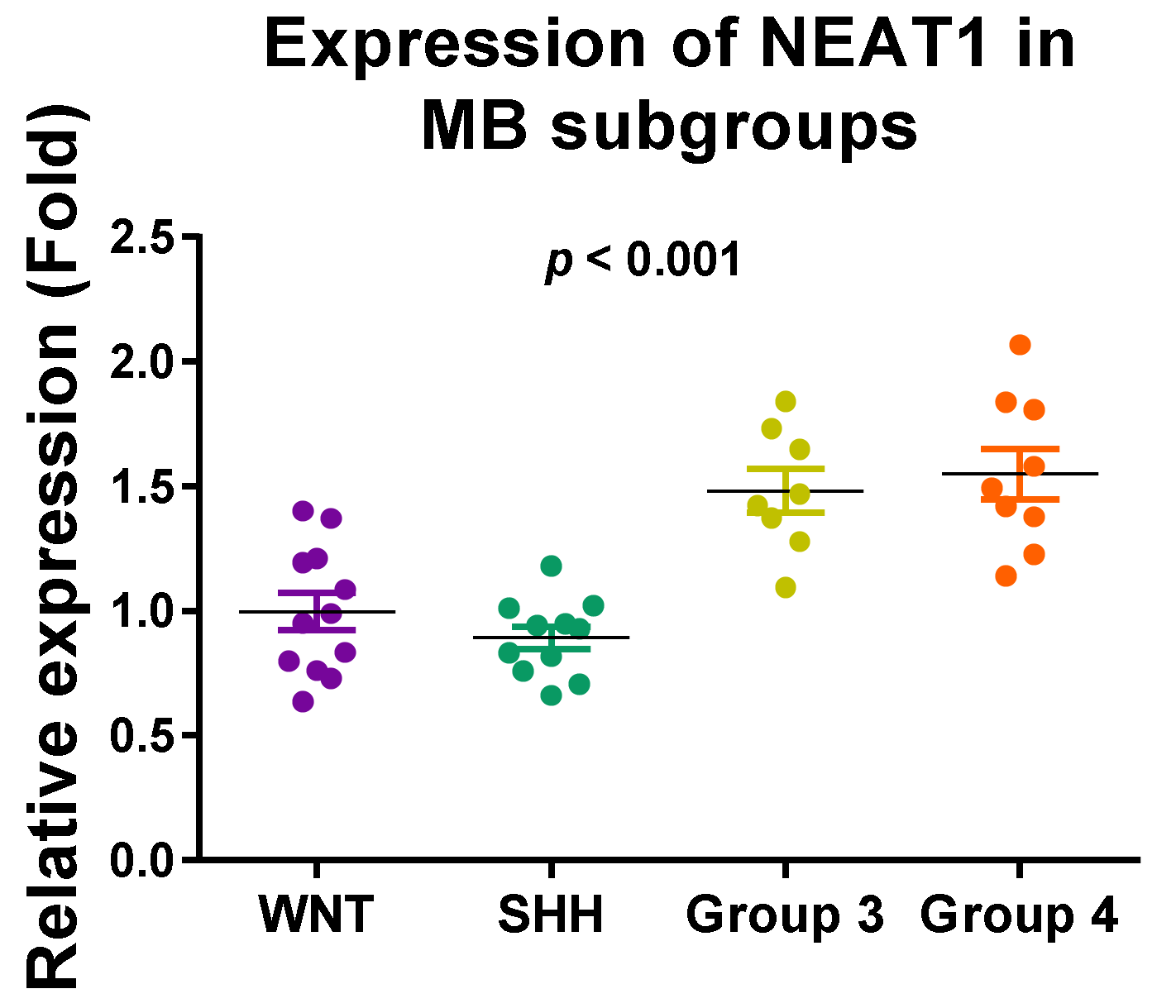

Supplement: Supplemental Material [file KBIE_A_2008695_SM2749.zip › supplementary/Figure S1.tif]

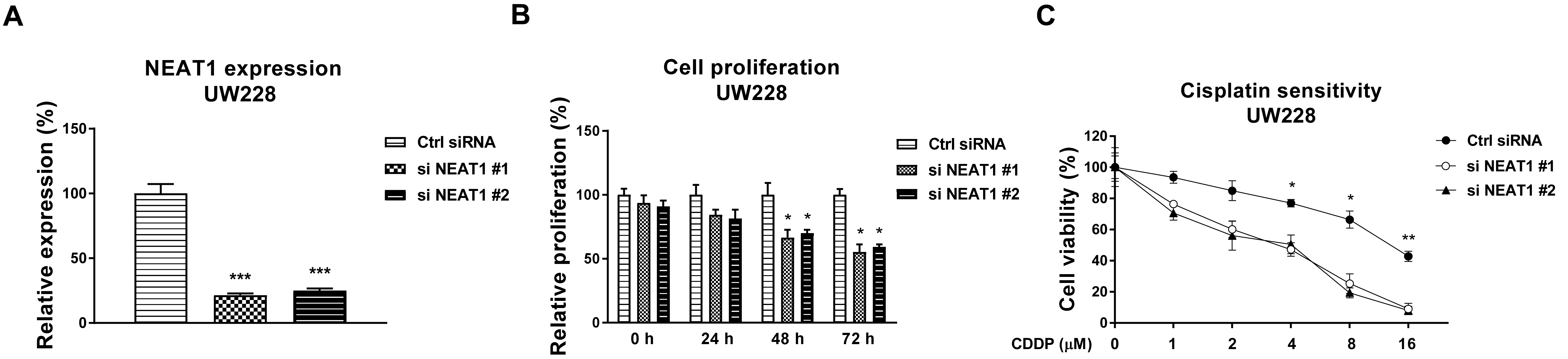

Supplement: Supplemental Material [file KBIE_A_2008695_SM2749.zip › supplementary/Figure S2.tif]

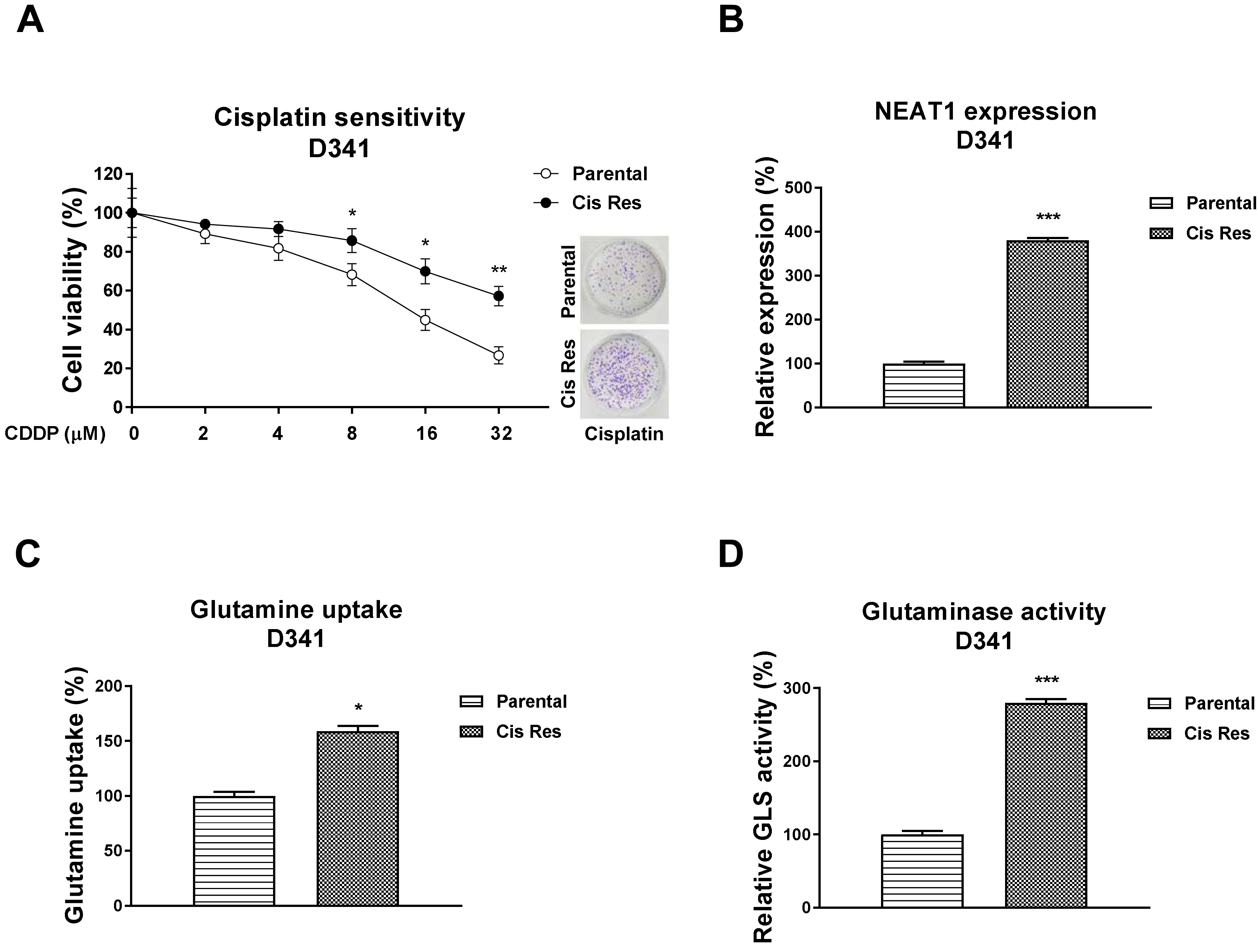

Supplement: Supplemental Material [file KBIE_A_2008695_SM2749.zip › supplementary/Figure S3.tif]

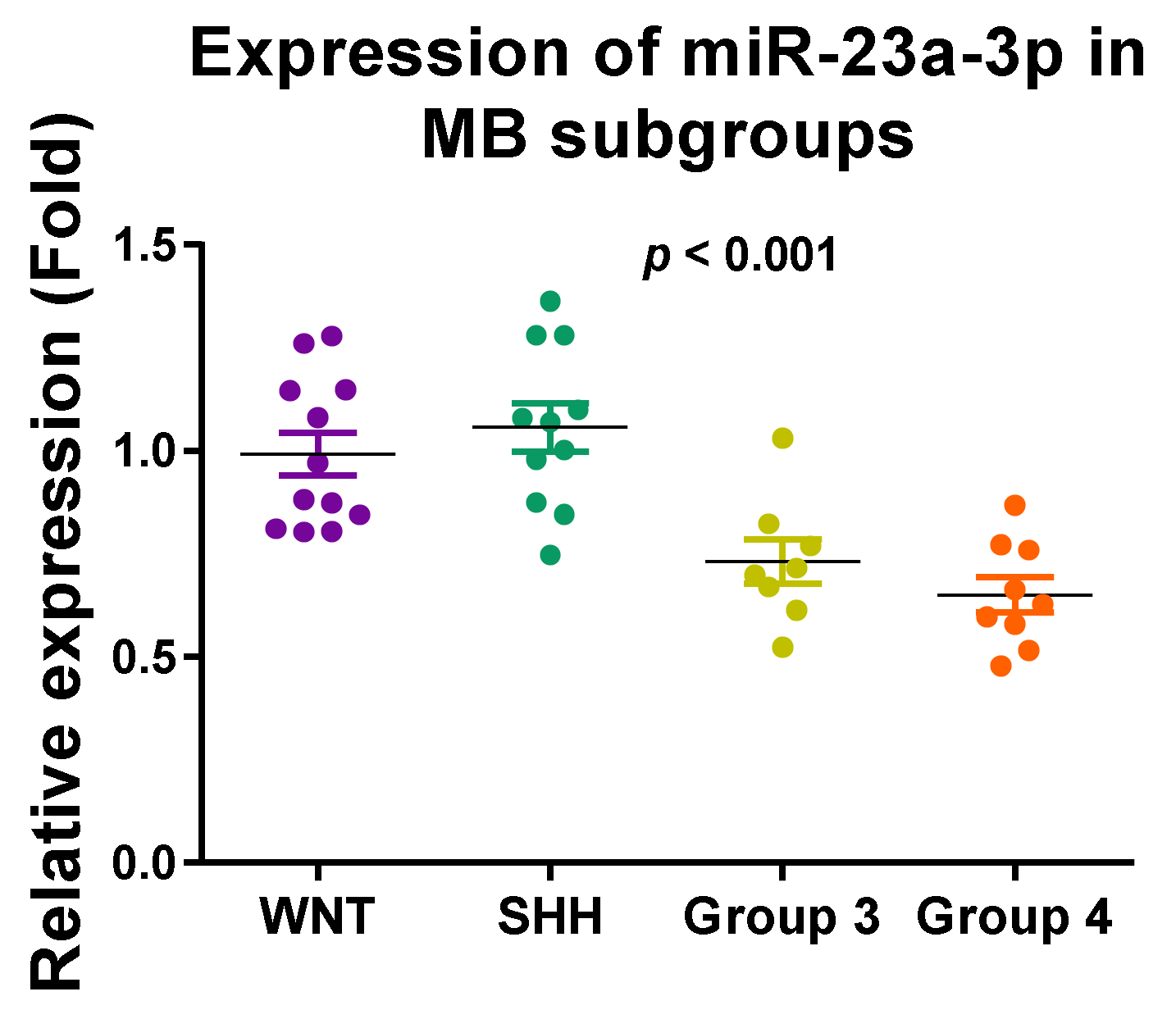

Supplement: Supplemental Material [file KBIE_A_2008695_SM2749.zip › supplementary/Figure S4.tif]

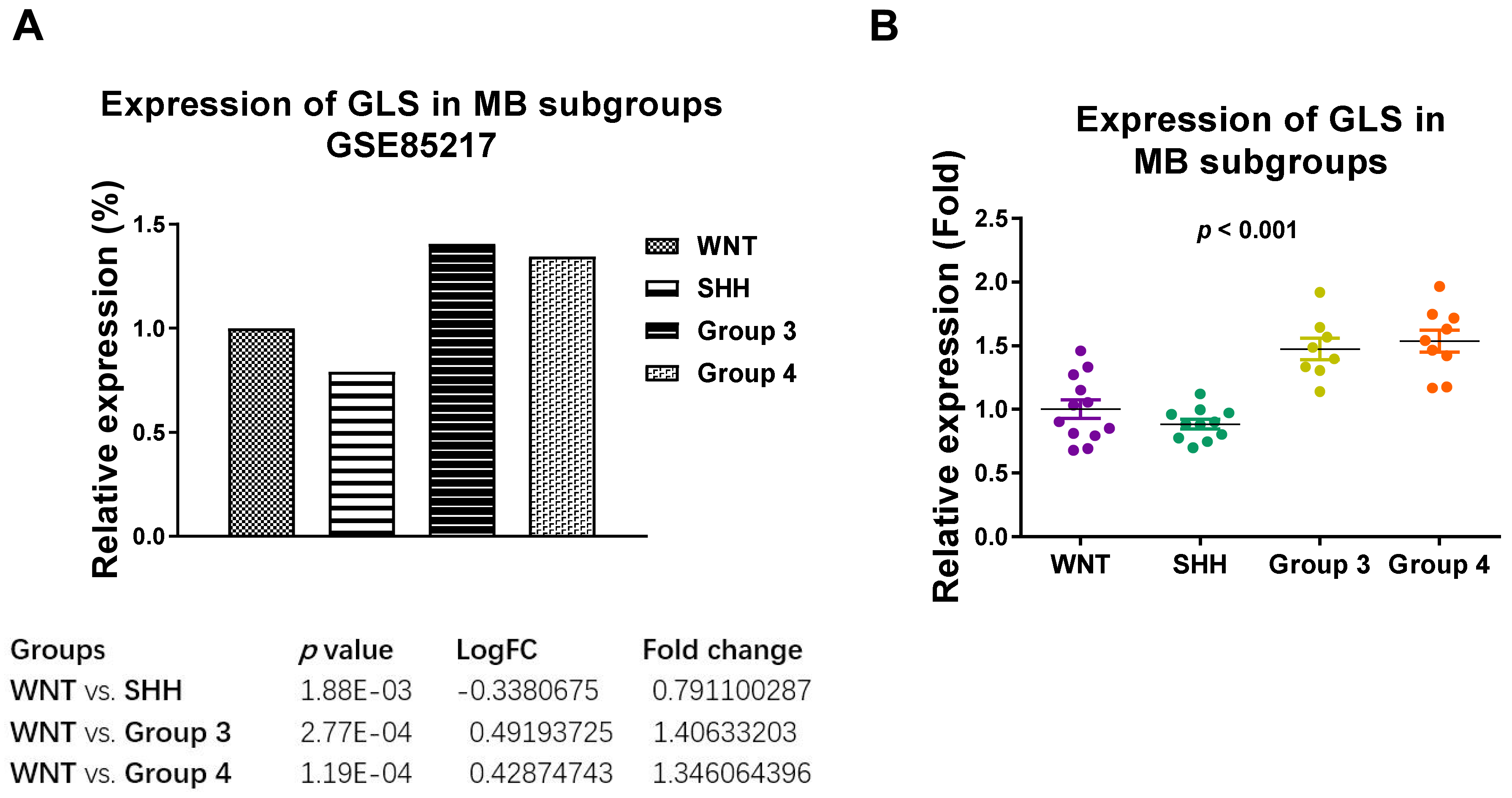

Supplement: Supplemental Material [file KBIE_A_2008695_SM2749.zip › supplementary/Figure S5.tif]

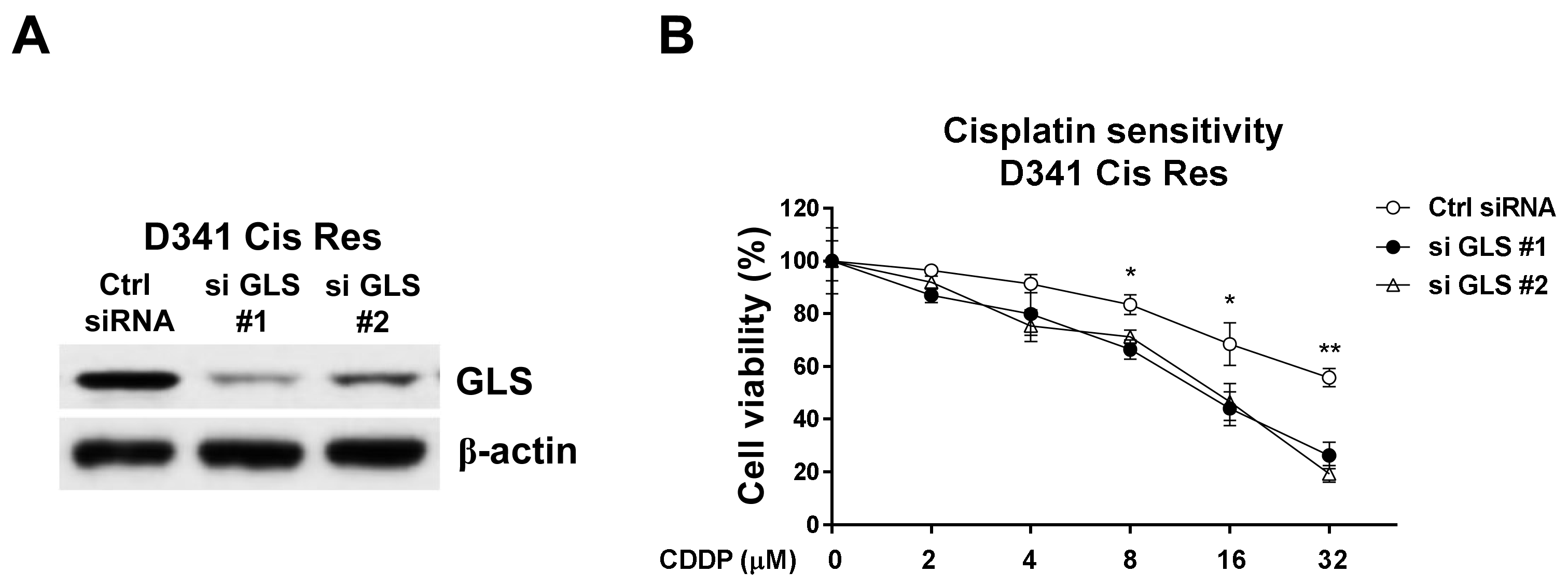

Supplement: Supplemental Material [file KBIE_A_2008695_SM2749.zip › supplementary/Figure S6.tif]

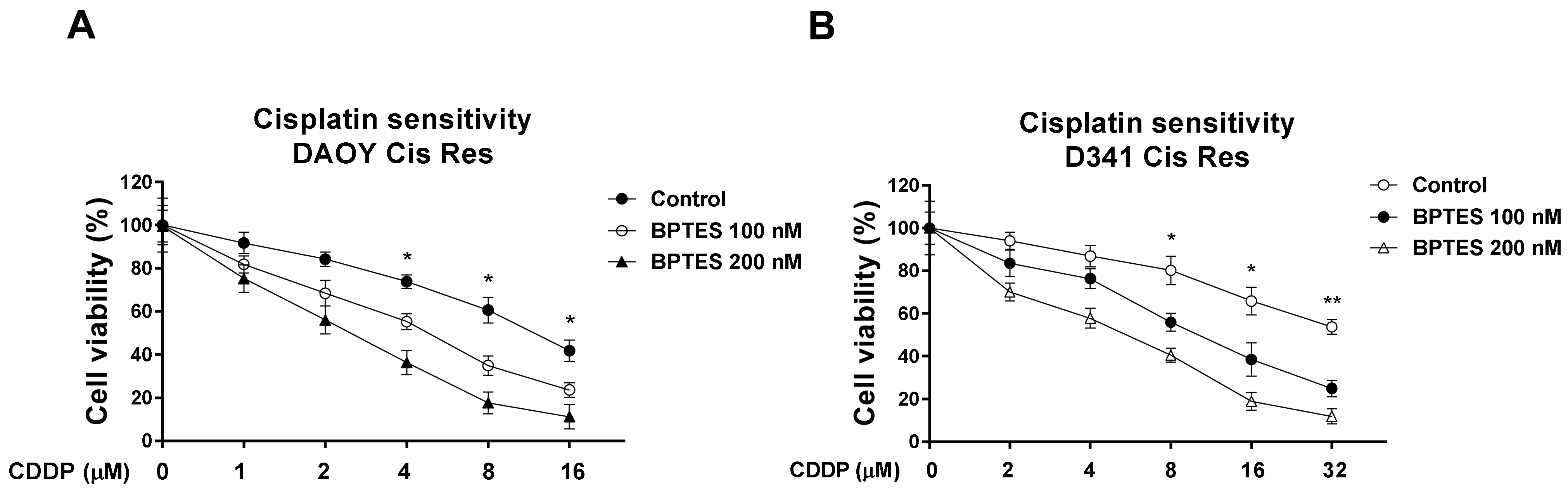

Supplement: Supplemental Material [file KBIE_A_2008695_SM2749.zip › supplementary/Figure S7.tif]
